# Synthesis and Anti-Bacterial Activities of Some Novel Schiff Bases Derived from Aminophenazone

**DOI:** 10.3390/molecules15106850

**Published:** 2010-10-08

**Authors:** Abdullah M. Asiri, Salman A Khan

**Affiliations:** 1 Chemistry Department, Faculty of Science, King Abdul Aziz University, P.O. Box 80203, Jeddah 21589, Saudi Arabia; 2 The Center of Excellence for Advanced Materials, King Abdul Aziz University, Jeddah 21589, P.O. Box 80203, Saudi Arabia

**Keywords:** Schiff bases, aminophenazone, antibacterial activity, ciprofloxacin

## Abstract

A series of 1,5-dimethyl-2-phenyl-1,2-dihydro-3*H*-pyrazol-3-one-containing Schiff bases were synthesized, characterized and screened for their antibacterial activities. The structures of the synthesized compounds were established by spectroscopic (FT-IR, ^1^H-NMR, ^13^C-NMR, MS) and elemental analyses. The anti-bacterial activities (with MIC values) of compounds were evaluated. The anti-bacterial screening results reveal that among the six compounds screened, four compounds showed moderate to good anti-bacterial activity. Among the tested compounds, the most effective compounds against four bacterial strains, viz. *Escherichia coli, Staphylococcus aureus, Salmonella typhimurium* and *Streptococcus pyogenes*, *are [(2-Chlorobenzylidene)amino]-1,5-dimethyl-2-phenyl-1,2-dihydropyrazol-3-one (4)* and *[(1,5-Dimethyl-3-oxo-2-phenyl-2,3-dihydro-1H-pyrazol-4-ylimino)methyl]benzonitrile(5)* with MIC values of 6.25 μg/mL*.*

## 1. Introduction

Compounds containing the -C=N- (azomethine group) structure are known as Schiff bases, usually synthesized from the condensation of primary amines and active carbonyl groups. Schiff bases are well known for their biological applications as antibacterial, antifungal, anticancer and antiviral agents [1,2]. Chloro and cyano groups containing Schiff bases at the C-2 position may display enhanced antibacterial effects [3,4]. Pyrazol-3-ones are found in numerous biologically active molecules recognized as having an important role in the animal and plant kingdoms. Different pyrazol-3-one-bearing compounds possess antibacterial [5], antifungal [6], antiinflammatory [7], antihypertensive [8], anti-HIV [9], antitumor [10], antifilarial [11] and anticonvulsant activities [12]. Recently, 4,5-diaryl-1H-pyrazole-3-ols were utilized as a versatile template for synthesizing compounds that act as potential cyclooxygenase-2 (COX-2) inhibitors and also show good selectivity for COX-2 versus COX-1 enzymes [13]. Some pyrazolones showed inhibition of TNF-α production in response to the tumor promotor TPA on HL-60 cells [4]. The pyrazol-3-one nucleus is known as an estrogen receptor ligand [14] and also as a novel class of antagonists for adenosine receptors [15]. Thus both the pyrazol-3-one nucleus and Schiff bases have attracted much interest in the development of pharmacologically active compounds. Since the pyrazol-3-one Schiff base moiety seemed to be a possible pharmacophore in various pharmacologically active agents, we decided to synthesize new pyrazol-3-one-containing Schiff bases as possible antimicrobial agents which might furnish better therapeutic results.

## 2. Results and Discussion

### 2.1. Chemistry

**Scheme 1:**
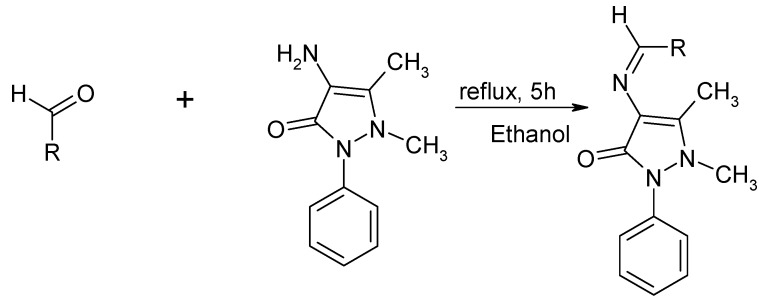
Synthesis of novel pyrazol-3-one derived Schiff bases.

In the present work, 1,5-dimethyl-2-phenyl-1,2-dihydro-3*H*-pyrazol-3-one Schiff base derivatives **1-6** were prepared by the reaction of 4-aminophenazone and the corresponding active aldehydes in accordance with the method described in the literature [16]. The synthetic route is outlined in Scheme 1. 

The chemical structures of the synthesized compounds were established by spectroscopic (FT-IR, ^1^H-NMR, ^13^C-NMR, MS) and elemental analyses. The FT-IR spectra of the pyrazol-3-one Schiff bases showed absorption bands at 2,830–2,940 cm^-1^ for aliphatic C–H and at 1,560–1,670 cm^-1^ for the azomethine group (–CH=N–). The nuclear magnetic resonance (^1^H-NMR) spectra of the compounds were recorded in CDCl_3_ and the structural assignments are given in Section 6. The 600 MHz ^1^H-NMR spectra of hydrazones 1-6 showed peaks of aromatic, methyl, and olefinic (–N=CH–) protons. These were all one proton singlets. The ^1^H NMR spectrum of Schiff bases (1-6) showed sharp singlet at δ 9.25–10.18 indicating the presence of azomethine (–CH=N–) proton. The sharp singlet at δ 3.11–3.22 indicated the presence of -CH_3_ group attached to the Nitrogen. The appearance of multiplets at δ 7.26–8.21 was due to aromatic protons. Moreover, the ^13^C-NMR spectra showed signals in the range of δ 109.14–111.75 ppm and at δ 134.61–135.28 ppm due to aryl and azomethine carbons, respectively. In the mass spectrum, compound **1** showed a peak at *m/z* 335 (M +1, 100%), which matches its molecular formula C_20_H_22_N_4_O. A peak at *m/z* 409 (M +1, 100%) was observed for compound **2** which is in conformity with the molecular formula C_26_H_24_N_4_O. Physicochemical data and elemental analysis results of the compounds are listed in Table 1. The spectral data of all the compounds are given in Section 4 .

**Table 1:** Physicochemical data of the synthesized compounds.

Compound no.	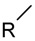	Molecular formula	M.p. ^o^C/ Crystallization	% Yield
1	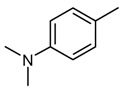	C_20_H_22_N_4_O	226/CHCl_3_	82
2	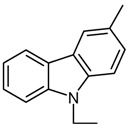	C_26_H_24_N_4_O	191/CHOH	76.5
3	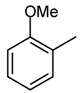	C_19_H_19_N_3_O_2_	222 /CH_3_Cl	76.8
4	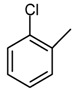	C_18_H_16_N_3_OCl	258 /CHCl_3_	78.5
5	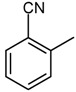	C_19_H_16_N_4_O	308/CH_3_OH	86.4
6	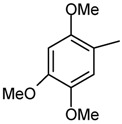	C_21_H_23_N_3_O_4_	381/CHCl_3_	72.8

### 2.2. Anti-bacterial activity

The anti-bacterial activity of the newly synthesized compounds **1-6** was evaluated against various pathogenic (Gram-negative and Gram-positive) bacterial strains viz., *Escherichia coli (E. coli), Staphylococcus aureus (S. aureus), Salmonella typhimurium (S. typhi), Streptococcus pyogenes* *(S. pyogenes).* The anti-bacterial activities were evaluated by the agar disc diffusion method as per the guidelines of the National Committee for Clinical Laboratory. Standards (NCCLS, 1997) [17]. The solvent used for the preparation of compound solutions (DMSO) did not show inhibition against the tested organisms (negative control).

The results of anti-bacterial screening of all the newly synthesized compounds are presented in Table 2. Most of the compounds showed moderate to good activity with MIC value in the range of 6.25 μg/mL in DMSO. Particularly, cyano and chloro derivative of Schiff base **(4&5)** showed good activity (zone of inhibition up to 19–28 mm at concentration of 6.25 μg/mL) against *Escherichia coli, Staphylococcus aureus, Salmonella typhimurium* and *Streptococcus pyogenes*. Compound 1 showed good activity against*, Staphylococcus aureus, Salmonella typhimurium* and *Streptococcus pyogenes*. (zone of inhibition up to 17–19 mm at concentration of 6.25 μg/mL). 

**Table 2:** Antibacterial activities of the compounds 1-6.

Product	*E. coli*	*S.aureus*	*S.typhinurium*	*S. pyogenes*
**1**	12 (25)	17 (6.25)	19 (6.25)	18 (6.25)
**2**	16 (6.25)	14 (25)	21 (6.25)	17 (6.25)
**3**	16 (6.25)	12 (25)	<10 (50)	<10 (50)
**4**	21 (6.25)	28 (6.25)	25 (6.25)	19 (6.25)
**5**	28 (6.25)	20 (6.25	26 (6.25)	22 (6.25)
**6**	15 (25)	13 (25)	<10 (50)	<10 (50)
**Ciprofloxacin**	32 (6.25)	23 (6.25)	28 (6.25)	24 (6.25)

MIC values are given in brackets. MIC (μg/mL) = Minimum inhibitory concentration, *i.e.* the lowest concentration of drug which completely inhibit bacterial growth. Ciprofloxacin was used as standard drug for anti-bacterial activity. Diameter of inhibition zone was measured in mm.

## 3. Experimental

### 3.1. General

All the chemicals and solvents used for this work were obtained from Merck (Germany) and Aldrich Chemical Company (U.S.A.). Melting points of the synthesized compounds were determined in open-glass capillaries on a Stuart-SMP10 melting point apparatus and are uncorrected. IR absorption spectra were recorded on a Shimadzu FTIR-8400s using KBr pellets in the range of 4,000–400 cm^-1^, ^1^H-NMR and ^13^C-NMR spectra were recorded on a JEOL AL600 FTNMR spectrometer operating at 600 MHz using. The ^1^H-NMR and ^13^C-NMR chemical shifts are reported as parts per million (ppm) downfield from TMS (Me_4_Si) used as an internal standard The splitting patterns are designated as follows; s, singlet; d, doublet; m, multiplet. Mass spectra were recorded on VG-AUTOSPEC spectrometer. IR, ^1^H-NMR, ^13^C-NMR and MS were consistent with the assigned structures. Elemental analyses (C, H, N) were done on a CHN Rapid analyzer. All the new compounds gave C, H and N analysis within ±0.03% of the theoretical values. Purity of the compounds was checked by thin layer chromatography (TLC) on Merck silica gel 60 F254 precoated sheets in chloroform/methanol mixture and spots were developed using iodine vapours/ultraviolet light as visualizing agents.

### 3.2. General procedure for the synthesis of Schiff Bases

A mixture of 4-aminophenazone (0.0058 mol, 0.5 g) and the corresponding active aldehyde. (0.0058 mol) in anhydrous methanol (15 mL) was refluxed at 80 ºC for 5 h with continuous stirring in the presence of few drop of acetic acid. Progress of the reaction was monitored by TLC. After completion of the reaction the solution was cooled. The heavy precipitate thus obtained was collected by filtration and purified by recrystallization from methanol and chloroform.

*4-[(4-Dimethylaminobenzylidene)-amino]-1,5-dimethyl-2-phenyl-1,2-dihydropyrazol-3-one* (**1**). C_20_H_22_N_4_O; IR *v*_max_ cm^-1^: 2893 (C-H), 1644 (C=C), 1656 (C=O), 1578 (C=N), 1133 (N-N); ^1^H-NMR (CDCl_3_) δ: 9.65 (s, 1H, CH olefinic), 7.78 (d, CHaromatic, *J =* 2.4 Hz), 6.72 (d, CHaromatic, *J =* 3.00 Hz), 7.26-7.48 (m, 5H, CHaromatic), 3.20, (s, N-CH_3_), 2.98 (s, N-CH_3_), 2.56 (s, N-CH_3_), 1.25 (s, CH_3_);^ 13^C-NMR (CDCl_3_) δ: 190.38, 161.31, 157.93, 151.87, 138.10, 135.05, 129.30, 129.06, 126.48, 125.87, 123.99, 122.80, 119.94, 111.81, 110.95, 40.24, 37.84, 10.24; MS (*m/z*, %): 335 (M+1, 45); Anal. Calc. for C_20_H_22_N_4_O: C**, **71.58; H, 6.48; N, 16.75, Found: C, 71.83; H, 6.63; N, 16.75.

*4-[(9-Ethyl-9H-carbazol-2-ylmethylene)-amino]-1,5-dimethyl-2-phenyl-1,2-dihydropyrazol-3-one* (**2**). C_26_H_24_N_4_O; IR *v*_max_ cm^-1^: 2976 (C-H), 1651 (C=C), 1675(C=O), 1566 (C=N), 1132 (N-N);^ 1^H-NMR (CDCl_3_) δ: 10.02 (s, 1H, CH olefinic), 8.67 (s, H3, CHaromatic), 8.24 (dd, H1, CHaromatic, *J =* 11.58 Hz), 8.13 (dd, H2, CHaromatic *J =* 12.72 Hz), 7.32-7.58 (m, 5H, CHaromatic), 4.47 (q, CH_3_-CH_2_-N, *J =* 10.74 Hz), 1.55 (t, CH_3_-CH_2_-N, *J =* 10.684 Hz ), 3.22 (s, N-CH_3_), 2.62 (s,-CH_3_); ^13^C-NMR (CDCl_3_)δ: 162.02, 158.53, 151.47, 143.56, 141.46, 138.10, 135.28, 134.97, 129.24, 129.04, 128.44, 125.91, 124.15, 123.16, 122.03, 120.80, 120.30, 119.36, 118.93, 109.14, 37.93, 37.71, 36.14, 13.86, 10.29; MS (*m/z*, %): 409 (M+1, 52); Anal. Calc. for C_26_H_24_N_4_O: C**, **76.45; H, 5.92; N, 13.92, Found: C, 76.35; H, 5.85; N, 13.82.

*4-[(2-Methoxybenzylidene)-amino]-1,5-dimethyl-2-phenyl-1,2-dihydropyrazol-3-one* (**3**). C_19_H_19_N_3_O_2_;IR *v*_max_ cm^-1^: 2830 (C-H), 1646 (C=C), 1691 (C=O), 1572 (C=N), 1135 (N- N); ^1^H-NMR (CDCl_3_) δ: 10.18 ((s, 1H, CH olefinic), 8.22(d, H3, CHaromatic, *J =* 2.58 Hz), 8.20 (dd, H4, CHaromatic, *J =* 11.22 Hz), 6.99 (dd, H5, CHaromatic, *J =* 12.42 Hz), 8.20 (d, H6 CHaromatic, *J =* 2.64 Hz), 7.39-7.56 (m, 5H, CHaromatic), 3.92 (s, O-CH_3_), 3.21 (s, N-CH_3_), 2.56 (s,-CH_3_); ^13^C-NMR (CDCl_3_) δ: 190.10, 160.92, 159.21, 153.55, 151.93, 134.90, 131.42, 129.09, 126.64, 126.38 125.90, 124.17, 120.44, 119.58, 113.08, 111.04, 55.48, 35.96, 10.16; MS (*m/z*, %): 322 (M+1, 58); Anal. Calc. for C_19_H_19_N_3_O_2_: C**, **71.01; H, 5.96; N, 13.07, Found: C, 70.85; H, 5.88; N, 12.98.

*4-[(2-Chlorobenzylidene)amino]-1,5-dimethyl-2-phenyl-1,2-dihydropyrazol-3-one* (**4**). C_18_H_16_N_3_OCl; IR *v*_max_ cm^-1^: 2939 (C-H), 1664 (C=C), 1678 (C=O), 1570 (C=N), 1132 (N-N), 718(C-Cl); ^1^H-NMR (CDCl_3_) δ: 9.71 ((s, 1H, CH olefinic), 7.79 (d, H3, CHaromatic, *J* *=* 1.80 Hz), 7.34 (dd, H4, CHaromatic, *J* *=* 1.20 Hz), 7.32 (dd, H5, CHaromatic, *J* *=* 1.2 Hz), 7.78 (d, H6 CHaromatic, *J =* 1.8 Hz), 7.35-7.50 (m, 5H, CHaromatic), 3.16 (s, N-CH_3_), 2.49 (s,-CH_3_); ^13^C-NMR (CDCl_3_) δ: 190.94, 160.72, 155.53, 152.04, 136.41, 135.87, 134.61, 130.93, 129.47,128.86, 127.05, 125.89, 124.48, 122.82, 118.31, 110.35, 37.85, 35.74, 10.24; MS (*m/z*, %): 326, 327 (M+1, 38, 56); Anal. Calc. for C_18_H_16_N_4_O: C**, **66.36; H, 4.95; N, 12.90, Found: C, 66.10; H, 4.85; N, 12.82.

*2-[(1,5-Dimethyl-3-oxo-2-phenyl-2,3-dihydro-1H-pyrazol-4-ylimino)methyl]benzonitrile* (**5**). C_19_H_16_N_4_O;IR *v*_max_ cm^-1^: 2940 (C-H), 1645 (C=C), 1672 (C=O), 1563 (C=N), 1139 (N-N); ^1^H-NMR (CDCl_3_) δ: 9.76 ((s, 1H, CH olefinic), 7.93 (d, H3, CHaromatic, *J* = 1.2 Hz), 7.39 (dd, H4, CHaromatic, *J =* 1.20 Hz), 7.35(dd, H5, CHaromatic, *J =* 7.2 Hz), 7.92 (d, H6, CHaromatic, *J =* 1.8 Hz), 7.69-8.21 (m, 5H, CHaromatic), 3.22(s, N-CH_3_), 2.51 (s,-CH_3_); ^13^C-NMR (CDCl_3_) δ: 190.08, 160.33, 154.05, 152.28, 141.98, 134.33, 132.32, 129.33, 129.05, 127.91, 127.41, 124.82, 122.84, 118.99, 117.84, 111.75, 35.48, 10.07; MS (*m/z*, %): 316 (M+1, 32); Anal. Calc. for C_19_H_16_N_4_O: C**, **72.14; H, 5.10; N, 17.17, Found: C, 72.08; H, 5.05; N, 17.08.

*1,5-Dimethyl-2-phenyl-4-[(2,4,5-trimethoxybenzylidene)amino]-1,2-dihydropyrazol-3-one* (**6**). C_21_H_23_N_3_O_4;_IR *v*_max_ cm^-1^: 2937 (C-H), 1644 (C=C), 1658 (C=O), 1591 (C=N), 1122 (N-N); ^1^H-NMR (CDCl_3_) δ: 10.02 (s, 1H, CH olefinic), 7.67 (s, H3, CHaromatic), 6.49 (s, H6, CHaromatic), 7.47-7.86 (m, 5H, CHaromatic), 3.93 (s, OCH_3_), 3.93 (s, OCH_3_), 3.84 (s, OCH_3_), 3.11(s, N-CH_3_), 2.48 (s,-CH_3_); ^13^C-NMR (CDCl_3_) δ: 188.07, 161.09, 154.92, 153.38, 151.31, 143.44, 134.99, 129.08, 128.89, 126.58, 124.10, 122.80, 117.23, 109.88, 96.88, 95.83, 56.70, 55.98, 37.85, 14.73, 10.27; MS (*m/z*, %): 382 (M+1, 52); Anal. Calc. for C_21_H_23_N_3_O_4_: C**, **66.13; H, 6.07; N, 11.02, Found: C, 65.95; H, 5.86; N, 10.93.

### 3.3. Antimicrobial activity assay procedure

#### 3.3.1. Disc diffusion method

The antimicrobial activity of newly synthesized compounds was evaluated according to the guidelines of the National Committee for Clinical Laboratory Standards (NCCLS, 1997) using the agar disc diffusion method [18]. Briefly, a 24/48 h-old culture of selected bacteria was mixed with sterile physiological saline (0.85%) and the turbidity was adjusted to the standard inoculum of McFarland scale 0.5 [~10^6^ colony forming units (CFU) per milliliter]. Petri plates containing 20 mL of Mueller Hinton Agar (MHA, Hi- Media) were used for all the bacteria tested. The inoculums was spread on the surface of the solidified media and Whatman no. 1 filter paper discs (6 mm in diameter) impregnated with the test compound (20 μL/disc) were placed on the plates. Ciprofloxacin (5 μg/disc, Hi-Media) was used as positive control for bacteria. A paper disc impregnated with dimethylsulfoxide (DMSO) was used as negative control. Plates inoculated with the bacteria were incubated for 24 h at 37 ºC and the fungal culture was incubated for 72 h at 25 ºC. The inhibition zone diameters were measured in millimeters. All the tests were performed in triplicate and the average was taken as final reading.

#### 3.3.2. Determination of MIC

Minimum inhibitory concentration (MIC) of any compound is defined as the lowest concentration which completely inhibits visible growth (turbidity on liquid media). MIC values were determined by testing performed according to the guidelines of NCCLS document M27-A [19]. Solutions of the test compounds, ciprofloxacin were prepared in DMSO at a concentration of 100 μg/mL. From this stock solution, serial dilutions of the compounds and ciprofloxacin (50, 25. 6.25 μg/mL) 50 (1 μL stock solution + 1 μL solvent), 25 (1 μL stock solution + 3 μL solvent), 6.25 (1 μL stock solution + 15 μL solvent), were prepared to determine the MIC. All determinations were done in triplicate and found the same result. The standard antibiotic, ciprofloxacin for bacteria was used as positive control and 100 μL of DMSO were used as a negative control. At the end of the incubation period, the MIC values were determined.

## 4. Conclusions

Some novel Schiff bases containing a pyrazol-3-one nucleus were synthesized by the reaction of 4-aminophenazone with the corresponding active aldehydes and were studied for their antimicrobial activity. The anti-bacterial screening results reveal that among all the compounds screened, compounds **1** and **2** showed moderate anti-bacterial activity, while compounds **4** and **5**, which bear chloro and cyano substituents, displayed good anti-bacterial activity (zone of inhibition up to 19–28 mm at concentration of 6.25 μg/mL) against *Staphylococcus aureus, Salmonella typhimurium* and *Streptococcus pyogenes* when compared with ciprofloxacin, used as standard. 

## References

[1] Nath M., Saini P.K., Kumar A. (2010). New di- and triorganotin(IV) complexes of tripodal Schiff base ligand containing three imidazole arms: Synthesis, structural characterization, anti-inflammatory activity and thermal studies. J. Organomet. Chem..

[2] Cheng L., Tang J., Luo H., Jin X., Dai F., Yang J., Qian Y., Li X., Zhou B. (2010). Antioxidant and antiproliferative activities of hydroxyl-substituted Schiff bases. Bioorg. Med. Chem. Lett..

[3] Mustafa I.M., Hapipah M.A., Abdulla M.A., Robinson T., Ward T.R. (2009). Synthesis, structural characterization, and anti-ulcerogenic activity of schiff base ligands derived from tryptamine and 5-chloro, 5-nitro, 3,5-ditertiarybutyl salicylaldehyde and their nickel(II), copper(II), and zinc(II) complexes. Polyhedron.

[4] Ciciani G., Coronnello M., Guerrini G., Selleri S., Cantore M., Failli P., Mini E., Costanzo A. (2008). Synthesis of new pyrazolo[5,1-c][1,2,4] benzotriazines, pyrazolo[5,1 c]pyrido[4,3-e][1,2,4] triazines and their open analogues as cytotoxic agents in normoxic and hypoxic conditions. Bioorg. Med. Chem..

[5] Upadhayaya R.S., Vandavasi J.K., Kardile R.A., Lahore S.V., Dixit S.S., Deokar H.S., Shinde P.D., Sarmah M.P., Chattopadhyaya J. (2010). Novel quinoline and naphthalene derivatives as potent antimycobacterial agents. Eur. J. Med. Chem..

[6] Sabaa M.W., Mohamed N.A., Mohamed R.R., Khalil N.M., Abd El Latif S.M. (2010). Novel quinoline and naphthalene derivatives as potent antimycobacterial agents. Carbohyd. Polym..

[7] Chandra T., Garg N., Lata S., Saxena K.K., Kumar A. (2010). Synthesis of substituted acridinyl pyrazoline derivatives and their evaluation for anti-inflammatory activity. Eur. J. Med. Chem..

[8] Kalla R., Zablocki J. (2009). Chapter 13 Recent Advances in Adenosine Receptor (AR) Ligands in Pulmonary Diseases. Annu. Rep. Med. Chem..

[9] Deacon S.W., Beeser A., Fukui J.A., Rennefahrt U.E.E., Myers C., Chernoff J., Peterson J.R. (2008). An Isoform-Selective, Small-Molecule Inhibitor Targets the Autoregulatory Mechanism of p21-Activated Kinase. Chem. Biol..

[10] Rostom S.A.F. (2010). Polysubstituted pyrazoles, part 6. Synthesis of some 1-(4-chlorophenyl)-4-hydroxy-1H-pyrazol-3-carbonyl derivatives linked to nitrogenous heterocyclic ring systems as potential antitumor agents. Bioorg. Med. Chem..

[11] Sujatha K., Shanthi G., Selvam N.P., Manoharan S., Perumal P.T., Rajendran M. (2009). Synthesis and antiviral activity of 4,4′-(arylmethylene)bis(1*H*-pyrazol-5-ols) against peste des petits ruminant virus (PPRV). Bioorg. Med. Chem. Lett..

[12] Rostom S.A.F., Ashour H.M.A., El-Razik H.A.A., El-Fattah A.E.H.A., El-Din N.N. (2009). Azole antimicrobial pharmacophore-based tetrazoles: Synthesis and biological evaluation as potential antimicrobial and anticonvulsant agents. Bioorg. Med. Chem..

[13] Patel M.V., Bell R., Majest S., Henry R., Kolasa T. (2004). Synthesis of 4,5-Diaryl-1H-pyrazole-3-ol Derivatives as Potential COX-2 Inhibitors. J. Org. Chem..

[14] Brana M.F., Gradillas A., Ovalles A.G., Lopez B., Acero N.,  Llinares F., Mingarro D.M. (2006). Synthesis and biological activity of *N*,*N*-dialkylaminoalkyl-substituted bisindolyl and diphenyl pyrazolone derivatives. Bioorg. Med. Chem..

[15] Fragkaki A.G., Angelis Y.S., Koupparis M., Tsantili-Kakoulidou A., Kokotos G., Georgakopoulos C. (2009). Structural characteristics of anabolic androgenic steroids contributing to binding to the androgen receptor and to their anabolic and androgenic activities: Applied modifications in the steroidal structure. Steroids.

[16] Gessi S., Sacchetto V., Fogli E., Merighi S., Varani K., Baraldi P.G., Tabrizi M.A., Leung E., Maclennan S., Borea P.A. (2010). Modulation of metalloproteinase-9 in U87MG glioblastoma cells by A_3_ adenosine receptors. Biochem. Pharmacol..

[17] Jarrahpour A.A., Zarei M. (2004). Synthesis of 2-({[4-(4-{[(E)-1-(2-hydroxy-3-methoxyphenyl) methylidene]amino}phenoxy)phenyl]imino}methyl)-6-methoxyphenol. Molbank.

[18] National Committee for Clinical Laboratory Standards (1997). NCCLS Approved Standard M27-A.

[19] Tuite J. (1969). Plant Pathological Methods, Fungi and Bacteria.

